# Synergistic Antimicrobial Activities of Chitosan Mixtures and Chitosan–Copper Combinations

**DOI:** 10.3390/ijms23063345

**Published:** 2022-03-20

**Authors:** Philipp Lemke, Lena Jünemann, Bruno M. Moerschbacher

**Affiliations:** Institute for Biology and Biotechnology of Plants, University of Muenster, 48149 Muenster, Germany; p_lemk01@uni-muenster.de (P.L.); l_juen03@uni-muenster.de (L.J.)

**Keywords:** chitosan, bioactivity, antifungal, copper fungicides, synergistic activity

## Abstract

Several recent studies revealed the significant contribution of intensive agriculture to global climate change and biodiversity decline. However, synthetic pesticides and fertilizers, which are among the main reasons for these negative effects, are required to achieve the high performance of elite crops needed to feed the growing world population. Modern agro-biologics, such as biopesticides, biostimulants, and biofertilizers are intended to replace or reduce the current agro-chemicals, but the former are often difficult to combine with the latter. Chitosans, produced from the fisheries’ byproduct chitin, are among the most promising agro-biologics, and copper fungicides are among the most widely used plant protectants in organic farming. However, the two active ingredients tend to form precipitates, hindering product development. Here, we show that partial hydrolysis of a chitosan polymer can yield a mixture of smaller polymers and oligomers that act synergistically in their antifungal activity. The low molecular weight (Mw) of this hydrolysate allows its combination with copper acetate, again leading to a synergistic effect. Combined, these synergies allow a 50% reduction in copper concentration, while maintaining the antifungal activity. This is potentially a significant step towards a more sustainable agriculture.

## 1. Introduction

Sufficient food production for a growing world population requires intensive agriculture, including effective plant protection measures. At the same time, the negative effects of the excessive use of chemical plant protectants on consumer health and the environment are increasingly apparent. To mitigate those by developing more sustainable agricultural practices, a reduction of chemical inputs is urgently required, but this needs to be accomplished without loss of efficacy. Despite their non-specific mode of action, copper-based fungicides are still in widespread use, pathogens do not develop resistance against them, and they are approved for use in organic agriculture. However, in the interest of a circular bioeconomy, the goal must be to reduce the copper-input in a field to an amount, which is later removed from the field with the harvest. To retain the antimicrobial efficacy at reduced dosage, copper fungicides may be combined with other active ingredients, ideally agro-biologics with no negative impact on the environment and for consumers. The agro-biologic with a potential for synergistic interactions with fungicides is chitosan. However, the combination of copper and chitosan is not trivial, as precipitates quickly form [1].

As an aminopolysaccharide consisting of *N*-acetyl-d-glucosamine (GlcNAc) units linked by β-1,4 glycosidic bonds, chitin is a frequent component of fungal cell walls and of the exo- or endoskeleton of many invertebrate animals. When some or all of the GlcNAc units in chitins are converted into d-glucosamine (GlcN) units via chemical or enzymatic de-*N*-acetylation, the resulting chitosans are polycationic molecules at slightly acidic pH values due to the protonation of the amino group. Unlike chitins, which form insoluble, crystalline fibers, chitosans are soluble at pH values below ca. 6, making them more attractive for a broad range of applications in diverse fields. Their non-toxicity towards animal and human cells, their biodegradability in the environment [2], as well as their antimicrobial [3,4] and plant strengthening activities [5,6,7] would appear to predestine them for agricultural use. However, despite their seemingly simple nature as a linear, binary copolymer, structure–function relationships of partially acetylated chitosans proved difficult to elucidate, making early chitosan-based products unreliable in the field. Fortunately, progress of the past two decades in the structural analysis of chitosans, followed by the development of quality-controlled production processes, have allowed crucial insights into structure–function relationships of chitosans [8,9]. As a result, products based on structurally well-characterized, ‘second generation’ chitosans are now increasingly appearing in the biomedical [10], but also in the agricultural sector [11].

To date, both antimicrobial and plant resistance inducing activities of chitosans are known to be strongly dependent on the physicochemical properties of chitosans, which in turn depend on their chemical structure. Structurally, chitosan molecules are characterized by three parameters. First, the degree of polymerization (DP) describes the number of monomeric units in a chitosan chain, and thus determines its length. The boundary between chitosan oligomers and polymers is not clearly defined, but is typically assumed around DP 20. Second, the fraction of acetylation (*F*_A_) describes the proportion of acetylated GlcNAc units within the molecule, and thus determines its charge density. Most of the commercially available chitosans have *F*_A_ values between 0.05 and 0.25. Together, DP and *F*_A_ determine the Mw of the chitosan. The third, but by far less studied structural parameter, is the pattern of acetylation (PA), which describes the sequence of GlcNAc and GlcN units within the molecule. All of the commercially available chitosans appear to have random PAs. While the influence of DP and *F*_A_ on antimicrobial and plant strengthening activities is now beginning to be understood, with chitosans of medium DP and low *F*_A_ having the strongest antimicrobial activities [12,13]., while a high DP and intermediate *F*_A_ is best to induce plant disease resistance reactions [14,15], the influence of PA is only just emerging [8,9,16].

However, similar to other polysaccharides and other polymers, chitosan samples are invariably mixtures of different chitosan molecules. Therefore, in addition to the three parameters described above, which define individual chitosan molecules, a chitosan solution is characterized also by its dispersity in DP, *F*_A_, and PA of the chitosan molecules of which it consists [17]. The only dispersity, for which a generally accepted method of analysis exists, is the DP (*Đ*). However, the influence of this parameter on biological activities of chitosan samples has been disregarded until very recently. It was found that a chitosan sample with high *Đ* had higher antimicrobial activities than expected when adding the antimicrobial activities of its constituents, apparently due to a synergistic effect of the chitosan oligomers and polymers contained in the sample [18].

Unfortunately, even the most efficient chitosan-based agro-biologics tend to perform well only under moderate inoculum pressure, when chitosan treatments often suffice to protect the crops from economically relevant losses, particularly in less intensive agricultural settings. However, in intensive Western-style agriculture, which requires optimal fertilization, water supply, and plant protection to realize the growth potential of high yielding elite varieties, the use of agro-biologics is often not sufficient, particularly in years with adverse climatic conditions. Currently, the potentially synergistic combination of agro-biologics with conventional agro-chemicals is emerging as a new strategy of integrated crop management, which promises to significantly reduce the input of chemicals in agriculture, even if not completely replacing them. This synergistic effect of structurally well-defined chitosans and synthetic fungicides has been well documented [13,19], while the combination of chitosan polymers with copper-based fungicides, though suggested early [20,21], has remained challenging due to the tendency to form precipitates. Possibilities to overcome this problem are the use of chitosan derivatives, such as carboxymethyl chitosan [22,23,24,25,26] or chitosan nanoformulations [1,25,27,28]. These strategies, which aim at harvesting synergistic activities of chitosans and other components, are pursued also for non-agricultural applications of chitosans, such as in cosmetics [29], skin regeneration [30], drug delivery [31] or material sciences [32]. However, both approaches are problematic from the registration point-of-view. In agriculture, these include potential health issues of respirable nanoparticles and the fact that chitosan derivatives, unlike chitosan itself, which is classified as a ‘basic substance’ in Europe and probably soon as a ‘minimum risk pesticide’ in the USA, require cost- and time-consuming environmental and consumer safety studies.

Our recent observation of a synergistic antimicrobial effect of small chitosan polymers and oligomers, which in combination exhibited even stronger effects than the most antimicrobially active polymers alone [18], may suggest an alternative to derivatization or nanoformulation, as low Mw chitosans can be expected to be more easily combined with copper than high Mw chitosans. Moreover, we chose a chitosan with a higher *F*_A_ than in our earlier studies, as chitosan–copper interactions increase with the decreasing *F*_A_. Similar to our earlier studies [1,18], we used the cereal pathogen *Fusarium graminearum*, the causal agent of head blight in wheat, as a difficult to control phytopathogen of significant economic relevance [33]. The overarching aim of our study is to contribute to the transition to a more sustainable agriculture, using the potential of the multifunctional agro-biologic chitosan to help in reducing the amount of agro-chemicals. Copper-based fungicides are still used in abundance, particularly in organic farming, where alternatives are often lacking. However, their long-term use has led to copper accumulation in agricultural soils, with potential environmental problems and, consequently, reductions in the legally accepted annual copper dosage. Over the past decades, the efficacy of copper-based products has been increased significantly, especially by decreasing the copper particle size. However, these efforts have lately met with a ‘glass ceiling’, where further improvements have not been achievable. Here, we show a 50% reduction in the copper concentration required for efficient fungal growth inhibition by synergistic combination with suitable chitosans.

## 2. Results

### 2.1. Structural Characterization of the Chitosan Samples

**Figure 1:**
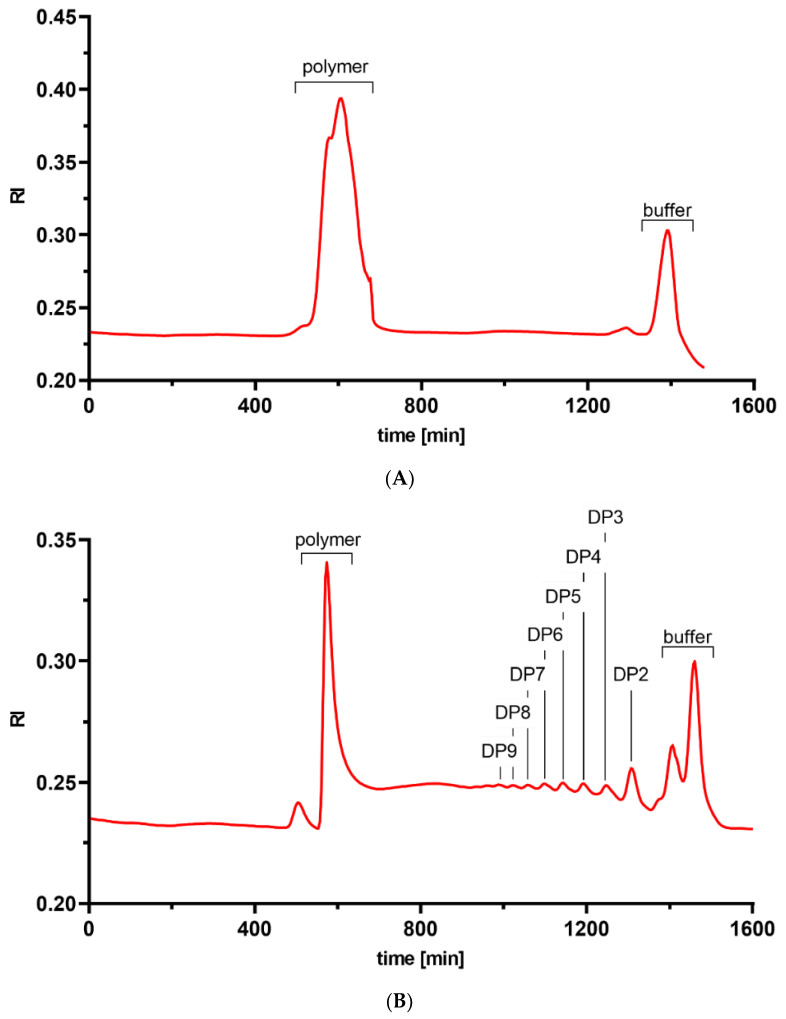
HP-SEC-RI-MALLS chromatograms of the parental chitosan (**A**) and the chitosan hydrolysate (**B**). The cut-off for separation of the polymer and oligomer fractions of the chitosan hydrolysate was made at 800 min, which corresponds to a DP of ca. 15.

The raw material used was a commercially available, microcrystalline chitosan (Mahtani Chitosan Pvt. Ltd. (Veraval, Gujarat, India)), which is produced from shrimp shell chitin using a mild, one-step, semi-homogeneous alkaline deacetylation process [34]. Using ^1^H–NMR spectroscopy and HP-SEC-RI-MALLS, we characterized the resulting chitosan (Figure 1A) as having an average *F*_A_ of 0.2 and a weight-average Mw of ca. 58.7 kDa (and thus a weight-average DP of ca. 350) with a *Đ* of 2.2 (Table 1).

To improve handling in the agricultural context [18], this parent chitosan (referred to as ‘chitosan polymer’) was partially chemically depolymerized by the same producer taking care to avoid concomitant deacetylation, yielding a less viscous chitosan hydrolysate which, at the same time, can be more highly concentrated (Figure 1B). Semi-prepSEC of this chitosan hydrolysate was used to separate an ‘oligomeric fraction’ of DP 2 to ca. 15 from a remaining ‘polymeric fraction’, which also had an average *F*_A_ of 0.2 and a weight-average Mw of ca. 43.3 kDa (DP ca. 250) with a *Đ* of 1.1 (Figure 1B). The oligomeric fraction represented ca. 25% of the dry weight of the chitosan hydrolysate.

### 2.2. Antifungal Activity

**Figure 2:**
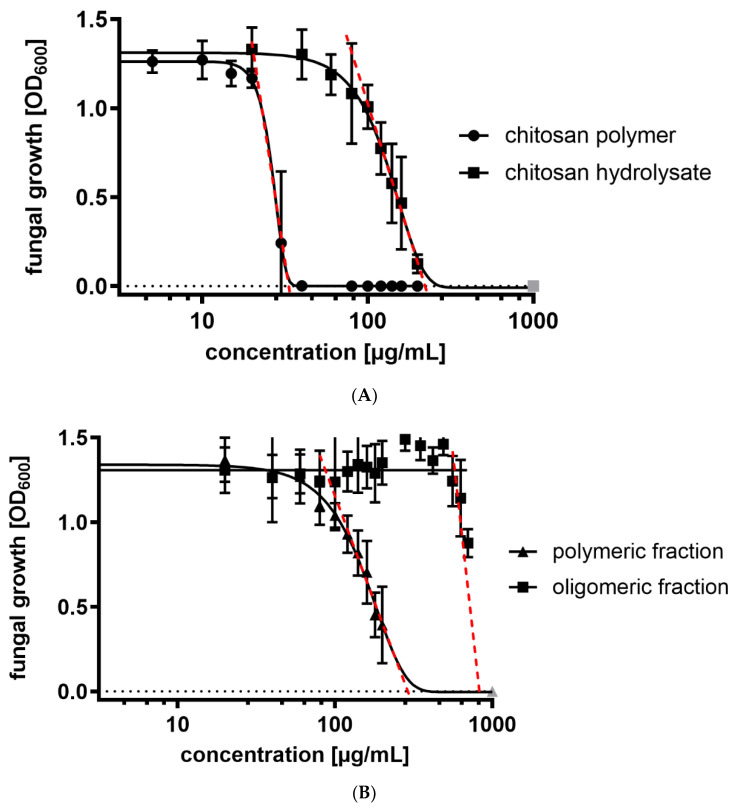
Antifungal activity against *F. graminearum* of (**A**) chitosan polymer (*F*_A_ 0.2, DP 347, *Đ* 2.2) and its chemical partial hydrolysate (*F*_A_ 0.2) and (**B**) the polymeric (*F*_A_ 0.2, DP 255, *Đ* 1.1) and oligomeric (*F*_A_ 0.2, DP 2-15) fractions of the hydrolysate. The IC_50_ values were calculated via nonlinear regression analysis using GraphPad PRISM software, for details see Materials and Methods. Artificial data points required by the program to achieve sigmoidal curves are marked in grey. Data shown are mean values ± SD of at least three independent experiments consisting of six replicates each.

The four chitosan samples were tested for their antimicrobial activity against the wheat pathogenic fungus *Fusarium graminearum* using a microtiter plate-based bioassay, where fungal growth is quantified over time based on the increase in optical density (Figure 2). Using chitosan concentrations ranging from 1 to 200 µg mL^−1^, we determined IC_50_ values for each chitosan sample, i.e., the concentrations at which fungal growth was inhibited by 50% compared to controls without chitosan (Table 2).

With an IC_50_ value of 26 µg mL^−1^, the chitosan polymer had by far the highest antifungal activity (Figure 2A). The chitosan hydrolysate and its polymeric fraction had higher IC_50_ values of 132 and 155 µg mL^−1^, respectively, i.e., they were less antimicrobial than the initial chitosan polymer. In concentrations up to 200 µg mL^−1^, the oligomeric fraction showed no antifungal activity (Figure 2B). When higher concentrations were tested, a slight growth inhibition was seen at concentrations beyond 500 µg mL^−1^, resulting in an estimated IC_50_ value of 739 µg mL^−1^. The observation of a higher antimicrobial activity of the chitosan hydrolysate compared to both its constituent polymeric and oligomeric fractions hinted at a synergistic effect of both fractions within the chitosan hydrolysate. This is particularly evident when considering that the oligomeric fraction, which constitutes 25% of the hydrolysate, is antimicrobially almost inactive and yet, its presence in the mixture leads to an antimicrobial activity, which is higher than the polymeric fraction alone.

### 2.3. Synergistic Antimicrobial Activity of Chitosan Oligomers and Polymers

**Figure 3:**
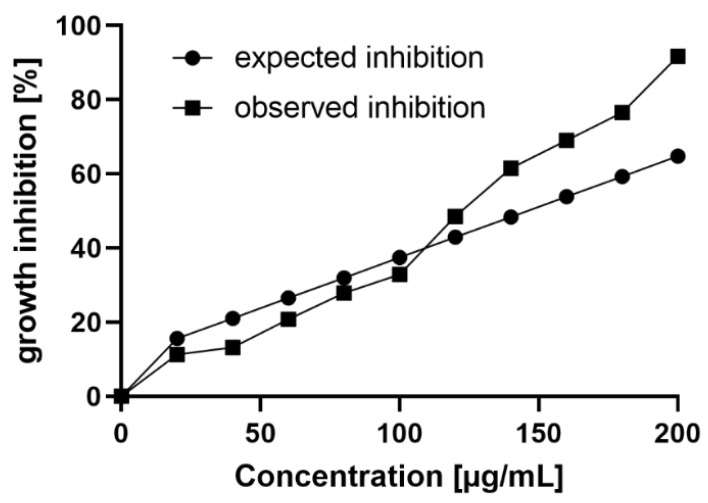
Expected and observed growth inhibition of the chitosan hydrolysate against *F. graminearum*. The observed growth inhibition is stronger than the expected growth inhibition at concentrations above 100 µg/mL.

To further elucidate the increased antifungal activity of the chitosan hydrolysate compared to what would be expected from the activities of its polymeric and oligomeric fractions alone, we calculated the amounts of polymers and oligomers in each hydrolysate concentration, then used the dose-response curves for both polymers and oligomers (see Figure 2) to determine the expected growth inhibition for these concentrations. The sum of oligomeric and polymeric inhibitions was plotted as the expected inhibition, which was then compared to the observed inhibition for the respective hydrolysate concentration (Figure 3). Clearly, the observed antifungal activity of the hydrolysate was stronger than the sum of activities of both polymers and oligomers at concentrations above 100 µg/mL. The strongest synergistic activity was observed at a concentration of 200 µg/mL, displaying a synergy factor (SF) of 1.4 when calculated using Abbott’s formula (Table S1).

### 2.4. Antimicrobial Activities of Chitosan Mixtures in Combination with Copper(II) Ions

**Figure 4:**
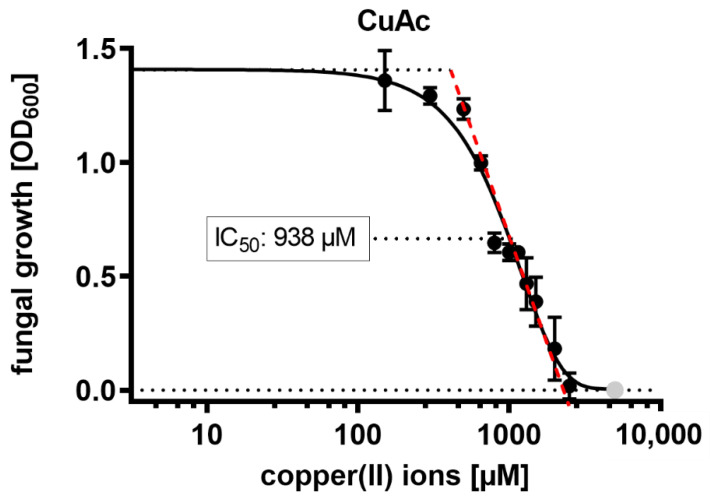
Antifungal activity of CuAc against *F. graminearum.* The IC_50_ value was calculated via nonlinear regression analysis of GraphPad PRISM software. The concentration values were log-transformed for both calculation and visualization. Artificial data points to achieve a sigmoidal curve are marked in grey.

Next, we combined the synergistically acting chitosan hydrolysate with the soluble salt copper acetate, which dissolves completely in water to yield the antimicrobially active copper(II) ion. The acetate salt was selected, as the chitosans were also solubilized using a 5% stoichiometric access of acetic acid. The IC_50_ value of copper(II) ions was around 1 mM or ca. 60 µg mL^−1^, i.e., in a similar range as the chitosans (Figure 4).

Next, we prepared three different mixtures of copper acetate with the chitosan hydrolysate, selecting concentrations based on the dose-response curves shown in Figure 2. As the goal of these combinations was to evaluate the potential of chitosan to reduce the amount of copper required for fungal growth inhibition, we used copper acetate at (i) a concentration close to its IC_50_ value (1000 µM), (ii) a still antimicrobially active, but 50% lower concentration (500 µM), and (iii) an almost inactive, again 50% lower concentration (250 µM). These decreasing concentrations of copper were combined with increasing, but low, and thus non-to-barely active concentrations of chitosan hydrolysate (20, 40, and 80 µg mL^−1^, respectively), as we were aiming for a synergistic effect of the two active ingredients. To account for the slightly differing vigor of the fungus in different experiments, which leads to difficulties in comparing absolute inhibitory rates, the two ingredients were also included at their different concentrations alone.

**Figure 5:**
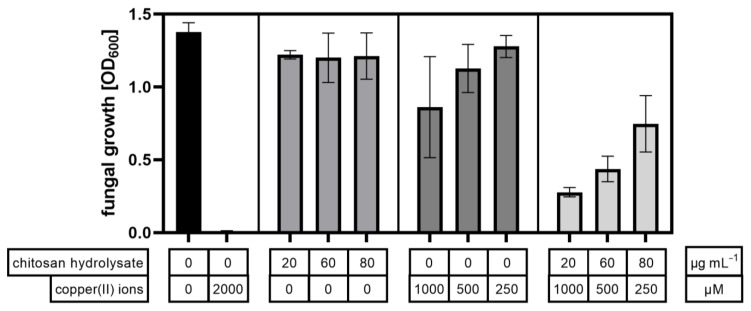
Growth of *F. graminearum* after 96 h in the presence of combinations of chitosan hydrolysate and copper(II) ions. In addition to the combinations, the individual compounds were tested in concentrations used for the combinations. Water and high copper(II) ion concentration (2000 µM) were used as controls.

In these experiments, both the copper(II) ions and the chitosan hydrolysate proved slightly less inhibitory against *F. graminearum* compared to the earlier experiments. Growth inhibition by copper showed a clear dose-dependency, but the highest concentration used exhibited only about 40% inhibition. All of the three chitosan concentrations used were equally and only slightly inhibitory, while all of the three copper–chitosan combinations showed significantly stronger inhibitory activities than the respective copper or chitosan concentrations alone (Figure 5). The highest antifungal activity, with a growth inhibition of ca. 80%, was seen with the highest copper concentration used (which in this experiment proved to be even lower than its IC_50_ value) combined with the lowest chitosan concentration used (which by itself was almost inactive).

Moreover, all of the three chitosan–copper combinations showed synergistic activity, with synergy factors ranging from 1.7 for the combination with the highest copper concentration to 2.5 for the combination with the lowest copper concentration (Table 3). Interestingly, the antifungal activity of the combination containing 1000 µM copper(II) ions and 20 µg mL^−1^ chitosan hydrolysate was not significantly different from the positive control containing 2000 µM copper(II) ions and no chitosan. Similarly, the combination of 250 µM copper(II) ions with 80 µg mL^−1^ chitosan hydrolysate was as antimicrobially active as 1000 µM copper(II) ions alone. Clearly, the addition of low amounts of chitosan hydrolysate allowed a strong reduction in copper concentrations without loss of antimicrobial efficiency.

## 3. Discussion

In this study, we verified that the chitosan treatment had direct antifungal effects on the vegetative growth of *F. graminearum*, and that the antimicrobial activity is dependent on the DP and *Đ* of the chitosan solution used. Chitosan is known to reduce the growth of this pathogen both in vitro [35] and in vivo [36], and to reduce the production of mycotoxins *in planta* [37,38]. Recent studies similarly demonstrated the control of other *Fusarium* species causing diseases of potato plants and tubers by the application of chitosan [39]. A plethora of studies have analyzed the dependency of antimicrobial activities of chitosans on their molecular weight, but a clear picture is still elusive. Most, but not all of the studies, conclude that chitosan polymers are more active than chitosan oligomers [12,33]. Our data shown here are in agreement with this hypothesis. The influence of Mw might differ between the fungi and bacteria, as well as between different fungal groups or Gram-positive and Gram-negative bacteria [12,32]. On the other hand, some authors claim that it is the target of action, such as germination versus mycelial growth, rather than the Mw, which determines its efficiency [40]. However, the discrepancies might also be caused by different definitions of ‘oligomers’, as most scientists seem to agree on a maximum size of ca. DP 15 for oligomers. However, some authors even include chitosans of DP 100 in this category, and by the use of chitosans with unknown, and possibly differing, degrees of acetylation [41]. Most likely, the most antimicrobially active chitosans are in this transition range of large oligomers to small polymers, i.e., in a DP range of ca. 20 to 50 [42].

The mode of action of the antifungal effect of chitosans is not entirely understood. However, it is clearly dependent on both the target organism, e.g., its cell wall or plasma membrane composition or its chitosanolytic potential [32,33,34], and the polycationic strength of the chitosan, i.e., its fraction of acetylation and the pH value of the medium [13,35,36,37]. These observations are compatible with both the assumption of extracellular interactions of chitosan and negatively charged cell wall and membrane surfaces, resulting in cell leakage and, possibly, cell death, as well as the possibility of intracellular interactions of chitosan with proteins or nucleic acids, which disturb the metabolic balance [38,39,40]. In fact, both assumptions may be correct, as chitosan polymers and oligomers are likely to have different modes of action. Chitosan polymers are unlikely to enter into cells [41,42], and thus most likely act extracellularly by disturbing the cell wall or membrane integrity [43]. This may facilitate transmembrane uptake of concomitantly present chitosan oligomers, which may exert intracellular effects, thus explaining the observed synergism between polymers and oligomers [18]. This dual mechanism would also explain the inactivity of small chitosan oligomers when applied alone, as they would fail to unfold their antifungal activity without previous membrane disruption by chitosan polymers.

While the synergistic antimicrobial effect of chitosan polymers and oligomers was originally shown for chitosans of *F*_A_ 0.1, herein, we describe it for chitosans of *F*_A_ 0.2, suggesting that it is a more general effect. The mechanism of this synergistic activity of chitosan mixtures is not yet understood. However, synergism is more likely to occur if the individual components of a mixture perform different modes of action on their targets. This tends to corroborate the above described mechanism of extracellular membrane disturbance by chitosan polymers, which leads to intracellular metabolic disturbance by chitosan oligomers. Even if we do not yet fully understand the mechanism of antifungal synergism of chitosan mixtures, the observation emphasizes the importance of considering the molecular weight dispersity (*Đ*) of a chitosan solution as a potentially crucial determinant of its biological activity [18].

Given that extracellular chitosanolytic enzymes that are present in a target tissue might slowly process chitosan polymers into oligomers, this synergistic activity might even play a role in the efficacy of chitosan polymers alone. If we know the optimal structural features required for the bioactivity of the membrane-targeting chitosan polymers and those of the intracellularly acting chitosan oligomers, and if we know the sequence- dependency of the chitosanolytic enzymes that are present in a target tissue, it might be possible to design the chitosan polymer in a way that it disturbs membrane integrity, and, at the same time, acts as a source for the slow release of the bioactive oligomers. Membrane disturbance apparently requires high charge density, thus a very low *F*_A_ is best [43]. However, this may lead to a small degree of degradation by chitinases and a large degree of degradation by chitosanases [44]. Clearly, the emerging possibility to control the pattern of acetylation might be a game changer in the development of designer chitosan polymers with optimized performance, taking into account their processing by sequence-dependent hydrolases in the target tissues [45].

Beyond synergism between chitosan oligomers and polymers, we found an additional synergism of the hydrolysate and copper(II) ions. The antimicrobial synergistic activity of chitosan and a second compound—mostly a fungicide—have been described repeatedly. For example, mixtures of chitosan and essential oils have been reported to inhibit anthracnose diseases in papaya [46] and mango in a synergistic manner [47]. Furthermore, chitosan was observed to synergistically enhance the activity of antibiotics against *P. aeruginosa* [48]. Other studies showed a synergistic effect of chitosan in combination with Fluconazole on the proliferation of yeast cells [49], as well as different *Candida* species [50]. Another emerging category of synergism studies is the usage of nanoformulated chitosan, which, for example, proved effective against the oomycete *Phytophthora capsici* [51] and fungi, such as *Botrytis cinerea* [52], *Neoscytalidium dimidiatum* [53], and *Fusarium*-caused tobacco root rot [54]. In a systematic study, chitosans ranging in size from oligomers to small polymers (DPn 9-206, *F*_A_ 0.15) were combined with five different commercially available fungicides, resulting in synergistic antifungal activity against *Botrytis cinerea*, *Alternaria brassicicola,* and *Mucor piriformis* in both in vitro and in vivo studies, as well as in field trials [19]. Moreover, in this case, the authors suggest that synergism is due to different modes of actions, e.g., through cell surface interactions of chitosan, which facilitate access of the fungicides to their intracellular targets.

**Figure 6:**
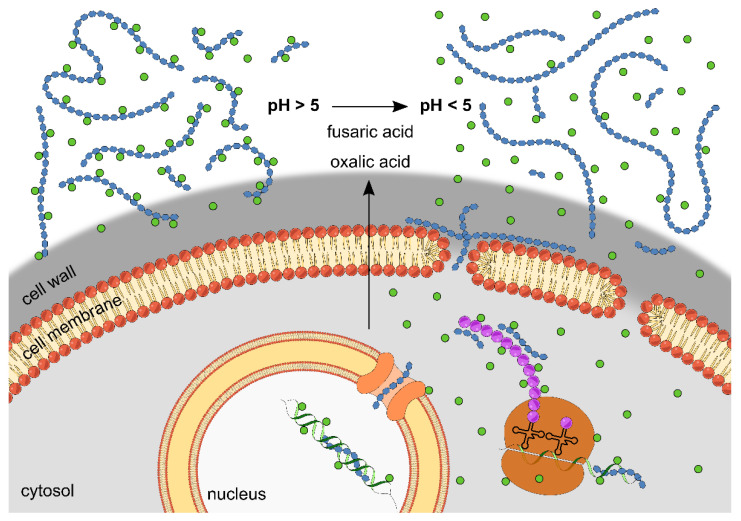
Potential mode of action of the synergistic antifungal activity of copper(II) ions and chitosans. The antifungal activity of copper(II) ions is based on ion uptake into the cell, followed by interaction with various chemical groups, ultimately disrupting the function of enzymes and other proteins, as well as nucleic acids. Chitosan polymers may be able to interact with or penetrate the fungal cell wall, which leads to destabilization of the membrane and uptake of chitosan oligomers into the fungal cell [18]. This also facilitates copper(II) ion uptake into the cell, explaining their synergistic action with membrane-permeabilizing chitosans. With a decrease in pH value by medium acidification, which is caused by the fungus, copper(II) ions increasingly dissociate from chitosan molecules, further enhancing the antifungal activity.

Copper salts are among the oldest fungicides. However, they are still in frequent use due to their high efficacy and low chance of tolerance or resistance development in target organisms. The mode of action of copper-based fungicides is believed to be based on copper(II) ion uptake, followed by interaction with various chemical groups, most prominently sulfhydryl groups, ultimately disrupting the function of enzymes and other proteins [55], which explains the synergistic action with membrane-permeabilizing chitosans (Figure 6). In our study, the pH value of the medium was around 5.5, where the adsorption capacity of chitosan for copper(II) ions is highest [1,56]. At lower pH values, the cationicity of the protonated amino function prevents copper binding, while at higher pH values, copper(II) ions interact with two amino functions, which lead to complexation and precipitation [57]. We assume that the fungus, by acidifying the medium, contributes to the dissociation of copper(II) ions from the chitosan, further increasing the antimicrobial activity.

This synergism between chitosan and copper has been previously reported. However, the formation of insoluble copper–chitosan complexes at higher pH values has long prevented the development of copper–chitosan products for plant protection, as copper exhibits phytotoxicity at the acidic pH value required for solubilization of the complexes. This problem can be overcome by nanoformulation, e.g., in the form of copper-loaded chitosan nanogels [1] or copper oxide nanoparticles [58], copper–silver core-shell nanoparticles [59] or titanium dioxide–copper nanoparticles [60] embedded in a chitosan matrix and forming chitosan–metal nanocomposites. However, the potential cytotoxicity of breathable nanoparticles renders their application in agriculture questionable. The synergy shown by combinations of copper and chitosan oligomer–polymer mixtures, which allows a significant reduction of copper dosage, may be a more practical approach, circumventing the need for nanoformulation. Given the major contribution of copper usage in agriculture to global environmental pollution [61], this development should contribute substantially to a more sustainable agriculture.

## 4. Materials and Methods

### 4.1. Preparation of Chitosan Solutions

A chitosan polymer solution was obtained by dispersion of chitosan powder (obtained from Mahtani Chitosan Pvt. Ltd. (Veraval, Gujarat, India) in dH_2_O and solubilization with a 5% molar excess of acetic acid relative to the free amino groups in the chitosan used. A partial acid hydrolysate of the chitosan was supplied by the same company as a 10% (*w*/*v*) solution. All of the solutions underwent sterile filtration through filters with a pore size of 0.22 μm before further usage.

### 4.2. Chitosan Characterization

Weight-average DP (DPw) of chitosan was determined using a combined system of high-pressure size exclusion chromatography coupled to refractive index detection and multi-angle laser light scattering analysis (HP-SEC-RID-MALLS), as described in [62]. From this, the dispersity *Đ* = *Mw*/*Mn* was calculated, with Mw as the weight-average and Mn as the number-average molecular weight.

To determine the *F*_A_ of chitosan, proton nuclear magnetic resonance spectroscopy (^1^H–NMR) was applied, according to a method described by [63]. This method uses the ratio between the integral of acetylated (i.e., methyl) group protons and the integral of GlcN protons with the formula *FA* = (1/3 × *I_CH_*_3_)/(1/6 × *I*(*H*_2_ − *H*_6_)), with *I*_*CH*3_ as the integral of methyl group protons and *I*(*H*_2_ − *H*_6_) as the sum of integrals of *H*_2_, *H*_3_, *H*_4_, *H*_5_, and *H*_6_ protons of GlcN [64].

### 4.3. Chitosan Fractionation

Separation of polymer and oligomer fractions in the chitosan hydrolysate was conducted using semi-preparative size exclusion chromatography (semi-prepSEC) with a SECurity GPC system (PSS Polymer Standards Service, Mainz, Germany), along with a set of three HiLoadTM 26/60 SuperdexTM 30 preparatory grade columns (2.60 × 180 cm) equilibrated with filtered and degassed elution buffer (150 mM ammonium acetate, pH 4.5) and a fraction collector (FRAC-200, Pharmacia, Uppsala, Sweden). For separation, a 5 mg mL^−1^ solution of the chitosan hydrolysate was passed through a 0.45-µm filter and loaded into a loop with a capacity of 5 mL. The elution flow rate was maintained at 0.6 mL min^−1^. The effluent was monitored with an online refractive index detector (Agilent 1200 series RIO, Agilent, Santa Clara, CA, USA) for around 27 h. After 8 h, fractions were collected every 10 min. After separating and collecting the desired fractions, the pooled fractions were lyophilized and re-solubilized in dH_2_O.

### 4.4. Cultivation and Induction of Conidia Production of Fusarium graminearum

Mycelium of *F. graminearum* strain DSM 4528 was cultivated and proliferated in petri dishes containing complete medium (CM) [65] agar (Supplementary Table S3). The plates were incubated in darkness, at 4 °C for storage or at 26 °C for vegetative growth induction. For conidia induction, mycelium precultured in CM was transferred to fresh CM medium containing carboxymethyl cellulose (CMM) [66] (Supplementary Table S4). After 8 days of shaking with 120 rpm at 26 °C in darkness, conidia were harvested via filtering the liquid culture through mesh or cotton. CMM was removed from conidia via centrifugation and re-suspension in dH_2_O.

### 4.5. Antifungal Activity

The antifungal activity of chitosan was measured in a 96-well microtiter plate, according to [67]. Accordingly, 10 μL of a spore suspension (7 × 10^3^ conidia per mL) or dH_2_O (blanks) was added to 150 μL CM, supplemented with 40 μL of a solution of chitosan, copper acetate (CuAc) or combinations of both compounds. The pH value of the medium was around 5.5. The plates were incubated under agitation as described above and fungal growth was recorded by UV–Vis spectrophotometric determination (Multiscan GO 60; Thermo Scientific, Waltham, MA, USA.) of the optical density at 600 nm (OD_600_) every 24 h for a total of 96 h. In the case that a dose-dependent antifungal activity of different chitosans was compared, the half maximal inhibitory concentration (IC_50_) values were determined via GraphPad PRISM software (GraphPad Software, Inc., San Diego, CA, USA). To achieve a sigmoidal curve, which is required for the calculation of IC_50_ values, artificial data points at very low and high concentrations had to be added, in some cases, to mark no or complete inhibition, respectively. These artificial data points are marked in grey in the respective figures if they fall into the concentration range of the *x*-axis shown. Statistical analysis was performed using one-way ANOVA and post-hoc Tukey test. All of the experiments were at least performed three times individually, with each experiment consisting of six technical replicates per agent.

### 4.6. Test for Synergistic Activity

To determine the potential synergy of chitosans and combinations of chitosan with CuAc, synergistic activity was calculated using Abbott’s formula, as follows:

*Cexp* = *A* + *B* − (*A* × *B*/100) (1)

where *Cexp* is the expected activity (in this case, inhibition of fungal growth), which is calculated from the activities of the individual components *A* and *B*. If the ratio (also known as synergy factor SF) of the experimentally observed activity (Cobs) and *Cexp* is greater than 1, the mixture shows synergistic activity, whereas a ratio close to 1 indicates an additive activity [68].

## 5. Conclusions

In this study, we corroborated the importance of both the molecular weight and the molecular weight dispersity of a chitosan sample for its antifungal activity. Partial hydrolysis of a chitosan polymer can yield a synergistically acting mixture of polymer and oligomer molecules. This mixture can be combined with copper(II) ions, generating another synergistic effect. On a fundamental research level, these observations suggest different modes of actions of chitosan polymers and oligomers, and of chitosans and copper. On an applied development level, our study suggests a way for the reduction of copper dosages required for efficient plant protection without the need for nanoformulation, which supports the transition to a more sustainable agriculture.

## Figures and Tables

**Table 1:** Chitosan analysis parameters.

Chitosan	*F*_A_	w.a. Mw (kDa)	w.a. DP	*Đ*
chitosan polymer	0.2	58.7	347	2.2
chitosan hydrolysate ^a^	0.2	n/a	n/a	n/a
polymeric fraction ^b^	0.2	43.3	255	1.1
oligomeric fraction ^b^	0.2 ^c^	n/a	2-15	n/a

^a^ The chitosan hydrolysate is derived from the chitosan polymer by partial chemical hydrolysis. ^b^ The polymeric fraction (75%) and the oligomeric fraction (25%) (*w/v*) are derived from the chitosan hydrolysate by semi-prepSEC. ^c^ The *F*_A_ of the oligomeric fraction was deduced to be 0.2, as both the hydrolysate and the polymeric fraction had a *F*_A_ of 0.2.

**Table 2:** IC_50_ values of the four chitosan samples against *F. graminearum*. Values are taken from the experiments shown in Figure 2.

Compound	IC_50_ (µg mL^−1^)
chitosan polymer	26
chitosan hydrolysate	132
polymeric fraction	155
oligomeric fraction	739

**Table 3:** Synergistic activity calculation via Abbott’s formula. The synergy factor SF is the ratio of the observed inhibition Cobs to the expected inhibition Cexp, with synergistic activity shown by SF > 1.

Combination	Chitosan Hydrolysate µg/mL	Copper(II) Ions µM	Cobs %	Cexp %	Synergy Factor Cobs/Cexp
1	20	1000	79.8	47.3	1.7
2	60	500	68.2	33.5	2.0
3	80	250	45.7	18.1	2.5

## Data Availability

The data presented in this study are available on request from the corresponding author.

## Supplementary Materials

The following are available online at https://www.mdpi.com/article/10.3390/ijms23063345/s1.
