# Supplementary material for: Synergistic Antimicrobial Activities of Chitosan Mixtures and Chitosan–Copper Combinations
Source: Int J Mol Sci. 2022 Mar 20;23(6):3345. doi: 10.3390/ijms23063345 (PMC8951000; doi:10.3390/ijms23063345)
Supplement: Supplementary file 1 [file ijms-23-03345-s001.zip › ijms-1597878-supplementary.pdf]

Table S1: Synergy calculations for the chitosan hydrolysate. The expected OD values were calculated using the known proportions of polymers (75%) and oligomers (25%) in the chitosan hydrolysate and the respective growth curves (**Figure 2**). The observed OD values were taken from the experimental data. Growth inhibitions were calculated via setting the OD_{600} value of the negative control to 100 % fungal growth, i.e., 0 % inhibition. The synergy factor SF is the ratio of the observed inhibition to the expected inhibition, with synergistic activity being assumed at SF > 1.

chitosan hydrolysate [μg mL^{-1}]	OD	% inhibiton (%obs)	polymer proportion [μg mL^{-1}]	OD	% inhibition polymer	oligomer proportion [μg mL^{-1}]	OD	% inhibition oligomer	inhibition sums (%exp)	SF (%obs / %exp)
0	1.5	0.0	0	1.5	0.0	0	1.5	0.0	0.0	-
20	1.3	11.3	15	1.4	3.9	5	1.3	11.6	15.6	0.7
40	1.3	13.2	30	1.4	9.3	10	1.3	11.7	21.1	0.6
60	1.2	20.8	45	1.3	14.7	15	1.3	11.8	26.5	0.8
80	1.1	27.9	60	1.2	20.1	20	1.3	11.8	32.0	0.9
100	1.0	32.9	75	1.1	25.5	25	1.3	11.9	37.5	0.9
120	0.8	48.5	90	1.0	30.9	30	1.3	12.0	42.9	1.1
140	0.6	61.5	105	1.0	36.3	35	1.3	12.0	48.4	1.3
160	0.5	69.0	120	0.9	41.7	40	1.3	12.1	53.9	1.3
180	0.4	76.5	135	0.8	47.1	45	1.3	12.2	59.3	1.3
200	0.1	91.7	150	0.7	52.5	50	1.3	12.2	64.8	1.4

Table S2: Individual growth inhibition potentials of all combination compounds. Growth inhibitions were calculated via setting the OD_{600} value of the negative control to 100 % fungal growth, i.e, 0 % inhibition. The growth inhibition values were required as observed efficiency values for Abbott's formula.

Compound	Cobs = growth inhibition
copper(II) ions 250 µM	7.1 %
copper(II) ions 500 µM	23.7 %
copper(II) ions 1000 µM	40.6 %
chitosan hydrolysate 20 µg mL^{-1}	11.3 %
chitosan hydrolysate 60 µg mL^{-1}	12.8 %
chitosan hydrolysate 80 µg mL^{-1}	11.9 %

Table S3: Complete medium for *F. graminearum* cultivation. This recipe provides liquid culture medium for *F. graminearum* cultures in flasks. For agar plates, 1.5 % (w/v) agar-agar was added. pH was adjusted to 5.8 with KOH and the medium was autoclaved before usage.

Component	Concentration
Yeast extract	0.1 % (w/v)
Casein hydrolysate	0.1 % (w/v)
Sucrose	1.0 % (w/v)
Tryptone	0.2 % (w/v)
Salt stock solution [1]	5.0 % (v/v)
Vitamin stock solution [2]	0.1 % (v/v)
Trace elements stock solution [3]	0.2 % (v/v)

[1] 10.4 g L^{-1} KCl, 10.4 g L^{-1} $MgSO_4 \cdot 7\ H_2O$, 30.4 g L^{-1} KH_2PO_4
[2] 0.5 g L^{-1} biotin, 16 g L^{-1} 4-aminobenzoic acid, 20 g L^{-1} pyridoxine hydrochloride, 50 g L^{-1} nicotinic acid
[3] 1 g L^{-1} $FeSO_4 \cdot 7\ H_2O$, 0.15 g L^{-1} $CuSO_4 \cdot 5\ H_2O$, 1.61 g L^{-1} $ZnSO_4 \cdot 7\ H_2O$, 0.1 g L^{-1} $MnSO_4 \cdot H_2O$, 0.1 g L^{-1} $(NH_4)_6Mo_7O_{24} \cdot 4\ H_2O$

Table S4: Carboxymethyl cellulose medium for *F. graminearum* conidia induction. Autoclaved before usage.

Component	Concentration
Carboxymethyl cellulose	1.50 % (w/v)
NH_4NO_3	0.10 % (w/v)
KH_2PO_4	0.10 % (w/v)
Yeast extract	0.10 % (w/v)
$MgSO_4 \cdot 7\ H_2O$	0.05 % (w/v)
